# Lack of Association between *NYD-SP18* Variant and Obesity. The Health Alcohol and Psychosocial Factors in Eastern Europe Study

**DOI:** 10.1159/000445982

**Published:** 2016-05-31

**Authors:** Jaroslav A. Hubacek, Hynek Pikhart, Ruzena Kubinova, Anne Peasey, Sofia Malyutina, Andrzej Pajak, Abdonas Tamosiunas, Martin Bobak

**Affiliations:** ^a^Center for Experimental Medicine, Institute for Clinical and Experimental Medicine, Prague, Czech Republic; ^b^Centre for Health Monitoring, National Institute of Public Health, Prague, Czech Republic; ^c^Department of Epidemiology and Public Health, University College London, London, UK; ^d^Institute of Internal and Preventive Medicine, Russian Academy of Medical Sciences, Novosibirsk, Russia; ^e^Department of Epidemiology and Population Studies, Institute of Public Health, Faculty of Health Care, Jagiellonian University Medical College, Krakow, Poland; ^f^Department of Population Studies, Institute of Cardiology of Lithuanian University of Health Sciences, Kaunas, Lithuania

**Keywords:** *NYD-SP18*, Polymorphism, Obesity, Body mass index, Waist-hip ratio, *FTO*, Energy intake

## Abstract

**Aim:**

To replicate the finding that the polymorphism rs6971091 within the *NYD-SP18* gene is associated with body mass index (BMI).

**Method:**

We analysed data of 29,284 adults (46.2% of males, mean age 58.9 (SD 7.3), mean BMI 28.6 (5.0 kg/m^2^)) examined within the Health Alcohol and Psychosocial Factors in Eastern Europe study in the Czech Republic, Poland, Lithuania and Russia.

**Results:**

BMI did not differ by rs6971091 genotype. In men, the mean BMI (SEs) in GG, GA and AA carriers were 27.8 (0.05), 27.9 (0.06) and 27.9 (0.14) kg/m^2^, respectively, (p = 0.26); in women, the corresponding values were 29.2 (0.06), 29.1 (0.07) and 29.1 (0.16), p = 0.57. In Czech subjects (n = 6,752), for whom the *FTO* rs17817449 genotype was available, there was no interaction between the *NYD-SP18* and *FTO* polymorphisms in determination of BMI. Adjustment for age, energy and fat intake and physical activity did not materially change the results. There was no association of the *NYD-SP18* genotype with waist-hip ratio.

**Conclusion:**

This study in a large Slavonic population sample suggests that the rs6971091 variant within the *NYD-SP18* gene is not an important determinant of obesity in middle-aged persons.

## Introduction

Obesity is a common preventable risk factor of a range of chronic conditions, including cardiovascular diseases, cancer and diabetes. In some populations, as much as 60% of individuals are overweight or obese [1]. Obesity or overweight generally results from positive energy balance caused predominantly by low physical activity and high energy intake, although other factors, such as sleeping deficit or social factors also play a role [2]. In addition to these environmental factors, obesity and body mass index (BMI) are significantly influenced by genetic factors. Twin studies suggest that genetic influences may explain as much as 40-70% of the variation in BMI [3]. However, despite intensive research efforts in the last decades, only a relatively small part of the BMI heritability has been attributed to specific genetic factors.

Among the polymorphisms with known association with obesity, the most important are the *FTO* 1st intron variants [4]. The study based on the NHLBI Family Heart Study and the Framingham study identified a single nucleotide polymorphism (rs697109) within the *NYD-SP18* gene (gene ID 84691) as associated with obesity [5]. The gene was identified using fine mapping of the region on chromosome 7, which has been known for a long time to be associated with obesity [6]. The authors reported an effect of the *NYD-SP18* variants on obesity that was even larger than the effect of the *FTO* polymorphism [5]. More recently, the association between the rs697109 SNP and BMI was confirmed in males, but not in females [7]. In addition, the additive effect between this SNP and 1st intron polymorphism of the *FTO* gene on BMI values has been detected [7]. Finally, a small intervention study in females suggested that *NYD-SP18* polymorphism could be a significant predictor of positive body composition changes after a short-term intensive life style intervention [8].

The *NYD-SP18* gene is known to play a role in the development of testicles (Locus ID 84691), but the mechanisms that may link it with obesity development are not known. Rs6971091 is a non-synonymous polymorphism (G>A; Lys242>Glu), and it was predicted that it could cause the misfolding of the native protein (http://snpeffect.vib.be/snp_main.php?id=27249604) [9].

To the best of our knowledge, the effect of the rs6971091 SNP on obesity has not been independently replicated in a different population sample. The aim of this study is to test the replicability of the previously described association between the rs6971091 SNP within the *NYD-SP18* gene and obesity-associated traits.

## Subjects and Methods

We analysed data from 4 large population-based cohorts of adults established as part of the Health Alcohol and Psychosocial Factors in Eastern Europe (HAPIEE) study in the Czech Republic, Lithuania, Poland and Russia [10]. The cohorts consist of random samples of both men and women aged 45-69 (45-75 in Lithuania) at baseline. Subjects were selected from population registers; no exclusion criteria have been applied.

Dietary intakes of energy and macro- and micro-nutrients were assessed by 143-items Food Frequency Questionnaire as described previously [11,12]. Physical activity was assessed by questions asking how many hours per week the responders engaged in sports, games or hiking. The socioeconomic status was characterised by participants’ highest achieved education and the economic activities they were involved in [10].

All subjects provided a written informed consent to participate in the study. The study was approved by appropriate institutional Ethics Committees in agreement with the Helsinki Declaration of 1975.

DNA was extracted from the peripheral blood and *NYD-SP18* SNP rs6971091 was genotyped using the KASP™ methodology (KBioscience, London, England) [13]. Genotyping call rate was 97.98%. Both in the entire study (p = 0.77) and within each of the 4 populations, there was no evidence of deviation from the Hardy-Weinberg equilibrium. Means and SEs of BMI and waist-to-hip ratio (WHR) were estimated by linear regression post-estimation of adjusted means. STATA statistical software (version 12) was used for all analyses. Age, energy intake, fat intake, physical activity, education and socioeconomic status were included in adjusted analysis. p values (calculated for trend) <0.05 were considered significant.

## Results

Descriptive characteristics of study participants are shown in table 1. There were only minor differences between countries. For instance, Lithuanians had the highest mean age and the highest mean BMI (both p values <0.001). The genotype (allele) distributions were similar across the countries; the overall allele distribution (with minor A allele frequency of 26%) were also similar to the distribution reported previously (frequency of the minor allele 23%) [5,7]. In our study, both overall and within each cohort, the rs6971091 polymorphism was not associated with BMI values; all p values were >0.45 in codominant models (table 1). The results remain insignificant after adjustment for age and other covariates and in dominant and recessive models. Results of WHR were similar; the mean (SEs) of WHR in males with GG, GA and AA genotypes were 0.943 (0.001), 0.943 (0.001) and 0.945 (0.002), respectively, with p = 0.432; in females, the corresponding values were 0.834 (0.001), 0.838 (0.001) and 0.835 (0.002), respectively, with p = 0.814.

We have not detected any significant interaction between energy intake and *NYD-SP18* polymorphism in predicting BMI, either in the combined male and female population or when males and females were analysed separately. We did not find any associations of this variant with total dietary intake of energy or saturated and unsaturated fats.

Finally, in the Czech cohort (n = 6,752), where data on *FTO* rs17817449 genotype were available [14], we examined an interaction between *FTO* and *NYD-SP18* genotypes (table 2). While *FTO* was strongly associated with BMI (as reported earlier [14]), there was no suggestion of an association between *FTO* and *NYD-SP18* polymorphisms (p = 0.323) or of an interaction between these 2 genotypes in determining BMI (p = 0.664) or WHR (p = 0.564, not shown in the table 2).

## Discussion

Genome-wide association studies and the deep sequencing of areas with high logarithm of odds score for obesity have detected dozens of SNPs with the potential to significantly affect BMI. However, many of the associations detected in the primary studies remain unconfirmed. It is important that results from even large genetic association studies are replicated in independent population samples [15]; the reasons include statistical issues (to exclude false positive findings), possible inter-ethnic differences and potential modification of genetic effects by environmental and life style factors.

There is a wide list of genes/variants potentially associated with BMI or other indicators of obesity; however, definitive conclusions are sometimes difficult to be drawn [16,17,18,19,20,21,22]. Importantly, not all genetic variants are associated with BMI under all conditions in all population, as is the case of *FTO*[23]. For several genotypes, it has been reported that dietary and genetics factors may interact with one another (e.g. variants at *MC4r*[24], *APOA5*[25] or *TFAP2B*[26]).

In the case of the promising candidate, SNPs within the *NYD-SP18* gene area, the potential physiological link between this gene and obesity remain unknown; however, this applies to some extent to most obesity-associated genes (such as *TMEM18* or *FTO*). In the original report [5], it was suggested that *NYD-SP18* polymorphism may influence plasma leptin levels.

The primary report [5] found the association with obesity in two independent population samples; however, the relatively small sample size in these studies (several hundred individuals in each sample) makes them prone to false positive results. More recently, the association has been detected in males, but not in females, in the Czech post-MONICA study [7]. The possible role of the *NYD-SP18* in the determination of BMI has been further suggested in a small intervention study [8] of overweight non-diabetic females; the results suggest that common homozygotes profit more from the intensive life style intervention than carriers of the minor A allele.

Our study, with almost 30,000 unrelated adults, was much larger. We have found not even a slight suggestion of a trend in BMI by *NYD-SP18*. In addition, in a subset of 6,500 individuals, we found no evidence of interactions with the *FTO* genotype or dietary intakes energy or fats.

There is one major limitation of our study, which can potentially explain the difference between the previous reports [5,7] and our study. Both previous papers [5,7] also included younger individuals, while the HAPIEE study only covers persons aged 45 years or older. We, therefore, cannot exclude the possibility that an association between the *NYD-SP18* genotype and BMI exists in younger age groups. However, given the results of this large study, we conclude that the rs6971091 SNP within the *NYD-SP18* gene is unlikely to be an important genetic determinant of BMI per se.

## Disclosure Statement

All authors declare no conflict of interest.

## Figures and Tables

**Table 1:** Basic characteristics of subjects with successful *NYD-SP18* genotyping

Country	Czech Republic	Lithuania	Poland	Russia	Total
Number	6,725	6,828	8,742	6,989	29,284
Males, %	46.0	46.0	49.0	43.1	46.2
Age, years	58.3±7.1	61.0±7.6	57.7±7.0	58.9±7.1	58.9±7.3
BMI, kg/m^2^	28.2±4.6	29.3±5.3	28.2±4.6	28.6±5.4	28.6±5.0
WHR	0.886±0.085	0.894±0.087	0.880±0.083	0.888±0.087	0.886±0.085

*NYD-SP18 rs6971091*					
GG	3,672 (54.6)	3,697 (54.1)	4,659 (53.3)	3,806 (54.5)	15,834 (54.1)
AG	2,582 (38.5)	2,645 (38.9)	3,482 (39.8)	2,703 (38.7)	11,412 (39.0)
AA	471 (7.0)	486 (7.1)	601 (6.9)	480 (6.9)	2,038 (7.0)
BMI by rs6971091 *NYD-SP18* in men					
GG	28.2±0.1	28.5±0.1	27.9±0.1	266±0.1	27.8±0.05
GA	28.5±0.1	28.5±0.1	28.0±0.1	26.5±0.1	27.9±0.06
AA	28.0±0.3	28.9±0.3	27.9±0.2	27.0±0.3	27.9±0.14
p value	0.59	0.45	0.80	0.46	0.26
BMI by rs6971091 *NYD-SP18* in women					
GG	28.1±0.1	30.2±0.1	28.4±0.1	30.2±0.1	29.2±0.06
GA	28.2±0.1	29.9±0.1	28.3±0.1	30.1±0.1	29.1±0.07
AA	27.7±0.3	30.1±0.3	28.4±0.3	30.2±0.3	29.1±0.16
p value	0.67	0.49	0.67	0.67	0.57

Genotype frequency is given as n (%), BMI values are reported as mean ± SE. Linear regression.

**Table 2:** BMI values (mean ± SEs) of by the combination of *FTO* (rs17817449) and *NYD-SP18* (rs6971091) polymorphisms in Czech subjects (both genders combined)

*NYD-SP18* genotype	*FTO* genotype				p for trend
	GG	GT	TT	total	
GG	28.6±0.2	28.3±0.1	27.8±0.1	28.2±0.1	<0.001
GA	28.8±0.2	28.3±0.1	28.0±0.2	28.3±0.1	0.002
AA	28.7±0.5	28.0±0.3	27.3±0.3	27.9±0.2	0.016
Total	28.7±0.1	28.3±0.1	27.8±0.1	28.2±0.1	<0.001
p for trend	0.458	0.434	0.851	0.828	0.662*

Mean BMI are reported as mean ± SE. Linear regression.

* p for interaction = 0.662.
